# Epilepsy care cascade, treatment gap and its determinants in rural South Africa

**DOI:** 10.1016/j.seizure.2020.06.013

**Published:** 2020-08

**Authors:** Ryan G. Wagner, Chodziwadziwa W. Kabudula, Lars Forsgren, Fredrick Ibinda, Lars Lindholm, Kathleen Kahn, Stephen Tollman, Charles R. Newton

**Affiliations:** a Studies of Epidemiology of Epilepsy in Demographic Surveillance Systems (SEEDS) – INDEPTH Network, Accra, Ghana; b MRC/Wits Rural Public Health & Health Transitions Research Unit (Agincourt), School of Public Health, Faculty of Health Sciences, University of the Witwatersrand, Johannesburg, South Africa; c Department of Epidemiology and Global Health, Umeå University, Umeå, Sweden; d Department of Clinical Science, Neurosciences, Umeå University, Umeå, Sweden; e KEMRI/Wellcome Trust Research Programme, Centre for Geographic Medicine Research – Coast, Kilifi, Kenya; f INDEPTH Network, Accra, Ghana; g Neurosciences Unit, UCL Institute of Child Health, London, United Kingdom; h Department of Psychiatry, University of Oxford, Oxford, United Kingdom

**Keywords:** Seizures, Anti-epileptic drugs, Treatment cascade, Healthcare

## Abstract

**Purpose:**

The percentage of people who are diagnosed with epilepsy (diagnostic gap), access, receive and adhere (treatment gap) to anti-seizure medication (ASM) in low- and middle- income countries remains low. We explored the epilepsy care cascade, measured the diagnostic and treatment gaps, and examined socio-demographic factors associated with adherence to ASMs in rural South Africa.

**Methods:**

Utilizing a population-based cohort of 311 people with active convulsive epilepsy (ACE) residing in rural northeastern South Africa, a questionnaire was administered to examine associations between demographic and socioeconomic factors and the epilepsy treatment gap. Blood was taken to measure levels of ASMs.

**Results:**

Of the 311 individuals diagnosed, 93 % of individuals reported being previously told they had epilepsy and 94 % reported previously attending a health facility for their epilepsy. ASMs were detected in 138 individuals (76 %) and optimal levels were detected in 67 individuals, resulting in a treatment gap of 63 % (95 % confidence interval [95 %CI]: 56 %–70 %). Self-reported specificity of ASM use was 23 % (95 %CI: 12–39 %) and individuals ≥18 years were significantly more likely to report taking ASM than children and were significantly (p = 0.011) more likely to be adherent.

**Conclusion:**

Most people with epilepsy in rural South Africa had been previously diagnosed with epilepsy and had accessed care for epilepsy, yet the level of ASM adherence remained low, significantly lower amongst children. Understanding ways of improving knowledge of and adherence to ASM in rural South Africa is necessary, especially amongst children. The epilepsy care cascade can be useful in identifying gaps in care and targeting interventions to reduce these gaps.

## Highlights

• Most people with active convulsive epilepsy in rural South Africa are diagnosed.
• Most individuals diagnosed have some blood level of anti-seizure medication.
• Most individuals do not have optimal levels of medication.
• The epilepsy treatment gap is high in rural South Africa, especially in children.
• Identifying the epilepsy care cascade may better allow for targeting of interventions.

## 1 Introduction

Epilepsy is a common neurologic disorder that currently affects more than 50 million people globally, with at least 80 % of cases found in low- and middle-income countries (LMICs) [1,2]. Whilst pharmacologic treatment results in seizure freedom in roughly 70 % of patients with epilepsy [3], the number of individuals diagnosed with epilepsy and receiving and correctly taking anti-seizure medication (ASM) in LMICs remains low. A 2014 review found that 59 % of people with epilepsy in sub-Saharan Africa do not receive any treatment and only 33 % of patients who do receive treatment are managed appropriately [4].

The epilepsy treatment gap (ETG), defined as the proportion of people with active epilepsy whose seizures are appropriately controlled over the total number of people with active epilepsy in a given population at a specific time [5,6], is found to be higher in both LMICs (compared to high-income countries) and particularly in rural areas (compared to urban areas), with significant intra-country heterogeneity [7]. Two systematic reviews found the ETG to be over 50 % in most LMICs [7,8]. A study from rural Kenya found the ETG to be 62.4 % (95 %CI: 58.1–66.6) [9], similar to findings from a small study from rural South Africa [10]. Whilst a potentially useful metric to compare access to and quality of epilepsy care across different contexts [6,11], the ETG does not provide an indication of the underlying factors responsible for the gap. Furthermore, the ETG tends to aggregate a number of steps that are necessary for an individual to receive adequate care for epilepsy. As such, using a more expanded model could allow researchers and clinicians to explore gaps in the care continuum.

**Fig. 1:**
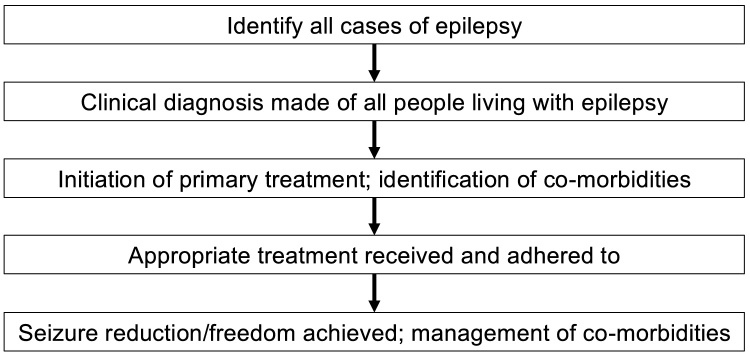
Proposed care cascade for people living with epilepsy.

The cascade of care model views care along a continuum: from the identification of possible cases, to diagnosis of a patient, accessing health care, initiating treatment, receiving appropriate treatment, through to disease resolution or acceptable management, including co-morbidities (Fig. 1) [12]. This approach resonates with the recently proposed stepped model of care found in the World Health Organization report on epilepsy [2]. The cascade of care model has been used in research on other chronic conditions, including depression [13], human immunodeficiency virus [14], and diabetes [15] and has recently been proposed to be used in epilepsy research as well [16]. The value in adopting this approach is the ability to identify where along the cascade patients fall off, thereby allowing researchers and clinicians to direct interventions at that specific stage of the care continuum. Furthermore, the cascade of care model takes a potentially broader approach in identifying possible factors responsible for the inadequate provision of epilepsy care. As Begley and colleagues suggest, understanding these underlying factors is vital to developing targeted interventions aimed at contextually specific need [11].

A number of potential structural factors have been suggested for the lack of provision of adequate care for epilepsy observed in LMICs, including lack of skilled healthcare providers, unavailability of ASMs at health facilities, inability to access health facilities and high cost of treatment [8]. These factors are likely to impact patients at different points along the care cascade. Additionally, misconceptions about the causes of epilepsy and fears of stigmatization are individual factors that may be associated with inadequate care [8]. Having learning difficulties, focal seizures and epilepsy for > 10 years were also found to be associated with non-adherence in Kenya [9].

Examination of potential causes of inadequate care provision requires consideration of both the characteristics of the healthcare system as well as those of the user. Andersen, in his original behavior model, articulated health-seeking behavior as a function of a number of domains, namely an individual’s predisposing factors (e.g. demographics, social structure, health beliefs), factors that either promote or impede use (e.g. availability of transportation) and perceived need for care (Table 1) [17]. Often researchers will examine enabling or impeding factors for utilization of healthcare services [18], such as affordability, availability and accessibility [19]; and neglect to examine the predisposing factors of the individual, such as demographic, social and belief structures that could influence adherence. Few studies, especially in LMICs, have explored whether social determinants, such as migratory status, education level, labor and socio-economic status and household structure and marital status, affect ASM adherence.

Utilizing a population-based prevalence cohort of people with active convulsive epilepsy in rural South Africa who took part in a series of cross-sectional surveys, we present the cascade of care for epilepsy, estimate the diagnostic and ETG in rural South Africa and explore individual and household level demographic and socioeconomic factors potentially affecting the treatment gap. Based on previous literature, we anticipate finding a comparably high ETG in rural South Africa and, specifically, a higher ETG than a diagnostic gap.

## 2 Methods

### 2.1 Study location

Located 500 km northeast of Johannesburg, the MRC/Wits-Agincourt Research Unit (http://www.agincourt.co.za) conducts population-based, health and socio-demographic research. The research unit, based in the Agincourt sub-district of rural Mpumalanga, South Africa, oversees a Health and Socio-demographic Surveillance System (HDSS) that covers roughly 120,000 individuals in 31 villages. Begun in 1992 and conducted annually, the HDSS census provides information on births, deaths and migrations as well as individual-level data on education, labor, socio-economic status (via household assets), household member structure, union status and distance to health facilities [20].

At the time this study was conducted, the Agincourt sub-district had a network of six public primary health care (PHC) clinics and one larger community health centre that provided health care to the population. Additionally, a public-private partnership community health centre within the sub-district provided primarily HIV and tuberculosis care. The PHC facilities refer patients to three government hospitals, which are situated 25−55 km from the site.

### 2.2 Participants

In a 2008 cross-sectional survey, we identified individuals within the Agincourt HDSS population with active convulsive epilepsy (ACE), defined as having ≥2 unprovoked convulsive seizures occurring more than 24 h apart and ≥1 seizure occurring in the 12 months preceding the study or currently taking ASM due to epilepsy [21]. The methods used to ascertain cases have been described previously [22], but briefly, a three-stage population-based survey was employed to identify all people with ACE. Two questions were added to the annual census and asked to every household respondent to identify individuals in the household who potentially had epilepsy. Individuals identified as potentially having epilepsy, were visited by a specially trained team of fieldworkers to ask additional questions about the history and presentation of the seizures. Individuals who had experienced at least one seizure within the last 12 months were seen by a study nurse who conducted a detailed clinical history and examination. All cases were reviewed and confirmed by the study neurologist (CRN).

### 2.3 Procedures

During the survey, blood was drawn and a detailed questionnaire, that included both clinical history and socio-demographic questions, was completed by all those who consented to take part in the study. In the case of children, parents or guardians were asked to respond on behalf of the child. During subsequent visits, information on treatment seeking behavior, including number of visits to health facilities and current ASM prescription was captured, as was seizure frequency. A poster board of locally available ASMs and their packaging was created and shown to patients to assist with recall of current and past ASM prescriptions.

### 2.4 Ethics

Written informed consent was sought from each participant in the study. Parental/guardian informed consent, along with participant assent, was sought in cases of children or cognitively impaired participants. Ethical clearance for the study was received from the Human Research Ethics Committee of the University of the Witwatersrand (M080455) and the Mpumalanga Province Department of Health’s Research and Ethics committee.

### 2.5 Analysis

#### 2.5.1 Care cascade & definition of adherence

The number of possible cases of ACE was estimated from the prevalence of ACE reported in a previous study from the same area [22]. Diagnosis of epilepsy, including who initially made the diagnosis and when, was self-reported.

Respondents were asked whether they were currently taking ASMs and which one(s). Blood samples were analyzed for the presence of phenobarbitone, phenytoin, carbamezepine and sodium valproate in the blood of those reported taking ASMs using a TDx FLx analyzer (Abott Laboratories, Abbott Park, IL, U.S.A.). The level of ASMs in the blood was also measured.

An individual was considered adherent if ASMs were detected in their blood, with detectable limits of 1.1 μg/mL (4.74 μmol/L) for phenobarbitone, 1.0 μg/mL (3.96 μmol/L) for phenytoin, 0.5 μg/mL (2.12 μmol/L) for carbamazepine and 1.0 μg/mL (6.93 μmol/L) for sodium valproate. Optimal ranges were defined as 10−40 μg/mL for phenobarbitone, 10−20 μg/mL for phenytoin, 4−12 μg/mL for carbamazepine [23], and 50−120 μg/mL for sodium valproate [24].

Sensitivity and specificity of the self-reported treatment gap were also calculated. The specificity was calculated by dividing the true negatives (those without detectable ASM levels who had reported not taking ASMs) by the sum of the true negatives and false positives (all those who had not reported taking ASMs). The sensitivity was derived by dividing the true positives (those who had reported taking ASMs and had detectable levels of ASMs in their blood) by the sum of the true positives and false negatives (or all those who had reported taking ASMs). Confidence intervals were calculated in Stata using the *diagt* command [25].

#### 2.5.2 Treatment gap determinants

Adapting Andersen’s behavioral model [17], we explored a number of potential determinants for the observed epilepsy treatment gap (Table 1). The variables were derived either from the cross-sectional survey (age, sex, employment, religion, union status, history of traditional medical use, seizure type and frequency, neurological deficits, learning difficulties, self-reported ASM use, duration of time with epilepsy, presence of burns and previous hospitalization); or from the annual Agincourt census data (ethnicity, current employment status, labor status, residency status, socio-economic status, distance to facility and kin availability, including mother’s availability).

To examine accessibility to the clinic, the Euclidian distance from the dwelling to the nearest health facility (clinic and hospital) was calculated. The socio-economic status, reported in quintiles, was determined using principle components analysis and dervied from self-reported household assets, whilst kin availability was simply defined as the number of co-residents within the dwelling as recorded in the census update.

### 2.6 Statistical analysis

Data collected within the HDSS was entered into a 2008 Microsoft SQL Server relational database management system (Seattle, WA, U.S.A.) and hosted within the field site. Data from the survey was entered into a MySQL database (OracleCorp, Redwood Shores, CA, U.S.A.). All data were imported and analyzed using Stata 14 (College Station, TX, U.S.A.). Individuals with missing data were excluded from the analysis. Logistic regression models were used to assess the association between each potential factor and the epilepsy treatment gap. Factors with p-values of <0.25 were included in a multivariate model. The odds ratio (OR) and the corresponding 95 % confidence intervals (95 %CI) were reported. As adherence was found to differ between adults (those aged 18 years and older) and children (those less than 18 years of age) in rural Kenya [26], a separate analysis was run for each - children and adults, with factors having a p-value of <0.05 in the multivariate model considered significant. Twenty-two factors were explored for adults and 21 for children.

## 3 Results

### 3.1 Prevalence and diagnosis

All 82,818 individuals that constituted the Agincourt HDSS population were screened for ACE, and an adjusted prevalence of 7.0/1000 individuals (95 %CI: 6.4–7.6) was determined after considering the sensitivity of the screening tool [22]. This prevalence estimate suggests that 580 individuals have ACE in the Agincourt HDSS. Of this estimated prevalence level, 311 (54 %) individuals with ACE were identified and interviewed, of which 159 (51 %) were males and 96 (31 %) were younger than 18 years old. The difference between the estimated number of cases of ACE (n = 580) and the number of actual cases identified (n = 311) is likely a consequence of migration, possible stigma and the low sensitivity (48.6 %) of the three-stage method used to identify cases [22,27]. Of those diagnosed with active convulsive epilepsy, 93 % had previously been told that they had epilepsy.

Of the 245 individuals (79 %) diagnosed with epilepsy who responded about their epilepsy diagnosis, 235 (96 %) believed they had epilepsy, whilst 7 (3 %) reported not having epilepsy and 3 (1 %) were unsure. Of these, 27 (11 %) had been diagnosed by a nurse at the public clinic, 91 (29 %) have been diagnosed by a doctor at the hospital, 34 (11 %) had been first diagnosed as part of this study, 85 (27 %) had been diagnosed by a traditional healer and 8 (3 %) were diagnosed initially by their church (n = 1), their mother or husband (n = 2), a teacher (n = 1) or self-reported as never being diagnosed (n = 4). More than half (54 %; n = 133) of individuals reported having 10 or more seizures in the year preceding the interview and 94 % reported having previously attended a health facility for epilepsy care.

### 3.2 Initial treatment

Of the 292 of individuals who responded regarding ASM use, 186 (64 %) reported currently taking ASM. Sixty-one individuals (33 %) reported being on polytherapy, whilst 98 individuals (53 %) reported being on monotherapy; 27 (14 %) could not name the ASM that they were taking (Table 2).

**Table 1:** Factors suggested to affect an individual’s health care utilization; adopted from Andersen’s behavior model [17].

Predisposing Factors	Enabling/Impeding Factors	Perceived Need
Age*	*Health System*	Duration of epilepsy*
Sex*	Distance to facility*	Type of therapy*
Ethnicity*	Cost to travel to facility	Seizure frequency*
Education level*	Perceived skill of staff	Seizure type*
Labor status*	Availability of ASMs	Neurological deficit*
Learning difficulties*		
Union Status*	*Personal/Family*	
Residency status*	Kin availability*	
Socio-economic status*	Mother/partner availability*	

* Examined in this study.

### 3.3 Adherence

**Fig. 2:**
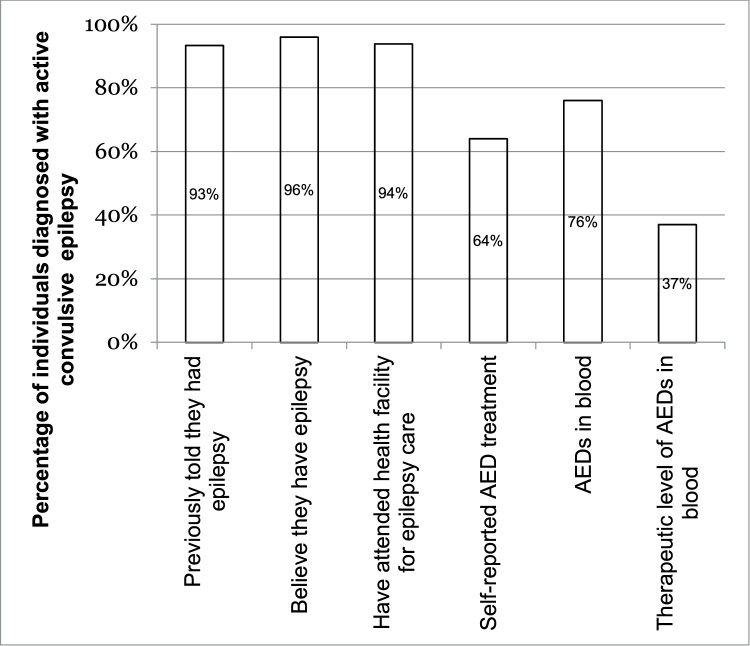
Care cascade for active convulsive epilepsy in rural South Africa.

Blood was drawn from 182 (59 %) of the 311 individuals interviewed, with the remaining participants either refusing to provide blood or blood unable to be attained. Neither age nor sex varied significantly between those who did and did not give blood. ASMs were detected in 138 (76 %) of the 182 individuals who gave blood. Optimal levels of ASMs were found in samples from 67 individuals (37 %) (Fig. 2).

### 3.4 Self-reported versus measured adherence

Of those who self-reported being on ASM, 81 (71 %) individuals had any ASM in their blood, whilst 50 (83 %) individuals who reported not taking ASM had ASM detected in their blood. This corresponded to a self-reported adherence sensitivity of 62 % (95 %CI: 53 %–70 %) and a specificity of 23 % (95 %CI: 12 %–39 %).

Adults were significantly (p < 0.001) more likely to report taking ASM than children (those < 18 years old) and were significantly more likely to be adherent (p = 0.011). No significant difference in either self-reported ASM use or adherence was found by sex in either children or adults. Overall, self-reported ASM use increased by age whilst adherence was highest in those aged 29−49 years (Table 3).

**Table 2:** Self-reported anti-seizure medication treatment for people with ACE- monotherapy and polytherapy.

ASM Drug Treatment	Self-reported
**Monotherapy**	**98**
Phenobarbitone	19
Carbamazepine	63
Phenytoin	7
Sodium Valproate	9
**Polytherapy**	**61**
Carbamazepine & Phenobarbitone	25
Phenytoin & Carbamazepine	18
Sodium Valproate & Carbamazepine	5
Phenytoin & Phenobarbitone	9
Phenobarbitone & Sodium Valproate	4
**Medication unknown to respondent**	**27**

### 3.5 Determinants

In adults, learning difficulties, presence of burns, number of seizure types, seizure frequency, number of years with seizures, co-resident with mother and distance to nearest primary health care facility were all found to have p-values < 0.25 and were included in the multivariate model exploring factors associated with self-reported ASM usage (Table S1). Only a higher seizure frequency (OR = 3.31; 95 %CI: 1.50–7.36; p-value 0.003) was found to be significantly associated with self-reported adherence. Number of years with seizures was borderline significant (OR = 1.49; 95 %CI 1.00–2.22; p = 0.052).

In the exploration of determinants associated with being adherent, as measured by ASMs detected in the blood, having more household members was significantly associated with a lower adherence (OR = 0.56; 95 %CI: 0.32−0.97; p = 0.040), though a number of variables did appear significant in the univariate analysis (Table S2).

For children, being >15 km from the nearest hospital was significantly associated with a lower self-reported use of ASM (OR = 0.15; 95 %CI: 0.03−0.80; p = 0.026), whilst greater seizure frequency (OR = 8.62; 95 %CI: 1.57–47.30; p = 0.013) and learning difficulties (OR = 6.64; 95 %CI: 1.07–41.01; p = 0.041) were significantly associated with self-reported ASM usage. A number of additional factors were significant in the univariate model (Table S3) but lost their discriminating power in the multivariate model.

**Table 3:** Reported ASM use (either self or by parents) and adherence by age.

Age band (in years)	Self-reported ASM use (95 %CI)	Adherent (measured as optimal ASM levels in blood) (95 %CI)
*0−5*	24% (9 %–49 %)	25 % (6 %–63 %)
*6−12*	26% (14 %–43 %)	11 % (3 %–34 %)
*13−18*	66 % (50 %–79 %)	32 % (18 %–51 %)
*19−28*	68% (56 %–79 %)	43 % (28 %–60 %)
*29−49*	75% (66 %–83 %)	45 % (34 %–58 %)
*50+*	77 % (62 %–88 %)	36 % (20 %–55 %)
*All ages*	64 % (58 %–69 %)	37 % (30 %–44 %)

No factors were associated with adherence in the children’s multivariate model, though closer distance to the nearest hospital, greater seizure frequency and previous hospitalization were all significantly associated with a higher adherence in the univariate model (Table S4).

## 4 Discussion

This study presents the cascade of care for epilepsy, in an effort to identify gaps in the convulsive epilepsy care continuum in rural South Africa. Using a three-stage, population-based study design, this study found 54 % of the estimated number of cases of ACE in the study area. Of those individuals, 93 % reported having previously been told that they had epilepsy. This suggests that at least within rural South Africa, the vast majority of people with ACE had been previously identified and told of their condition, which is the first step in the care cascade. This figure may be higher than found in this study as some individuals may have forgotten being told that they had epilepsy or misunderstood.

Interestingly, nearly one-third of individuals with epilepsy were first diagnosed by a traditional healer. There are many traditional healers in the communities in which this research took place and previous research has shown traditional healers to ‘treat’ a wide range of conditions, including epilepsy [28,29]. Further research on how rapidly an individual is diagnosed with epilepsy after their second seizure and by whom is needed. This would provide a better understanding of the timing between having epilepsy (usually considered after someone has a second seizure [30]) and when they are diagnosed. Earlier diagnosis can lead to earlier ASM initiation, in most cases resulting in a reduced seizure frequency and improved quality of life.

Interventions to direct the referral of possible cases of epilepsy from traditional healers to local biomedical health care facilities is also warranted. Referrals from traditional healers to health care facilities exist for other conditions, such as HIV, in similar settings [31] and referring individuals suspected of having epilepsy to health care facilities would allow for confirmation of an epilepsy diagnosis and initiation on ASM. Furthermore, involving traditional healers in treatment support and monitoring may provider culturally relevant psychology support, positively impact on adherence and ultimately reduce the ETG.

Of those who had been previously told that they had epilepsy, 178 (64 %) reported being on treatment for their seizures. This is lower than a study from rural Kenya, which found that 74 % of individuals diagnosed with ACE reported being on treatment [8]. Interestingly, whilst this is lower, within the current study, a number of individuals who reported not being on treatment had detectable levels of ASMs in their blood. Not surprisingly, and similar to the same study from rural Kenya [8], this study found poor specificity in self-reporting of ASM use and suggests additional instruction and education about why they are receiving ASM and the importance of adhering to it should be given to patients by health care providers when receiving their ASMs and, ideally, then followed up in the community. Educational programs have been suggested as a way to reduce stigma and improve adherence; however, a one-day health education program in rural Kenya did not improve adherence, though it did improve knowledge of epilepsy and its causes [32]. This suggests that such an intervention may improve the specificity of self-reported ASM use due to increased knowledge about epilepsy, but not the level of adherence. Designing behavioral interventions, such as intensive reminder or implementation intention interventions, may be more effective at improving adherence [33].

The percentage of those found with detectable levels of ASMs in this rural South African study was 76 % (95 %CI: 69 %–82 %) of those whose blood was sampled. This is much higher than rural Kenya, were ASMs were only detected in in 38 % of blood samples tested [9]. This finding may be a result of ASM being available to patients free of charge in public health care facilities, as ASMs form part of the Essential Drugs List in South Africa. This differs from many other African countries where the costs of ASM can be prohibitive and have been found to contribute to the observed ETG [9]. In some ways, South Africa provides a natural experiment, with the results suggesting that providing ASMs to people with epilepsy ‘free of charge’ is not the only intervention needed to reduce the treatment gap. Although ASMs are ‘free’, a recent study from Agincourt has highlighted other, potentially prohibitive, out-of-pocket costs, such as transportation costs, to be associated with seeking care for epilepsy at both clinics and hospitals [28].

Optimal levels of ASMs are significantly lower in children, which confirms previous studies from rural Kenya that have found lower adherence amongst children [9,32]. This is of considerable concern since complications, including convulsive status epilepticus have been found to be more common in younger ages [34,35]. Furthermore, adolescence is known to be challenging in terms of drug adherence [36]. Lower adherence and subsequent repeated seizures are linked to poorer educational attainment and lower quality of life, both serious considerations, especially for younger individuals. That said, further research is needed to develop and test interventions targeting the improvement of ASM adherence amongst children.

Factors of perceived need, including number of years with epilepsy and seizure frequency (in adults) and seizure frequency (in children), were found to be significant when examining factors associated with self-reported ASM use.

### 4.1 Limitations

This study was designed to explore the level of and adherence to ASMs as a way to estimate the epilepsy treatment gap. However, while undertaking the work, the concept of an epilepsy care cascade evolved. As such, the study provides data on a number of stages of the care cascade, but due to its design, does not completely evaluate the proposed epilepsy care cascade. Further studies should be designed and undertaken to evaluate the entire epilepsy care cascade as well as examine the outcomes of the treatment (which was not formally done in this study).

To estimate the overall prevalence, and therefore, the number of people living with active convulsive epilepsy in the study site, the analysis relied on the methodological sensitivity reported in a similar study that took place in Kenya [27]. This approach, given contextual differences, may result in a different sensitivity in rural South Africa to that of Kenya.

With regards to measured ASM levels, the current study only drew blood to measure ASM levels once and did not collect information on the timing of medication ingestion, which would have helped to explore the pharmacokinetics of the drugs. As a result, it is possible that individual variability in drug elimination rates and blood ASM concentration half-lives may have resulted in higher or lower levels of antiepileptic drugs compared to others. Furthermore, this study did not ask about concomitant medication usage, which may have also impacted the measured ASM levels. As such, repeating this study and including the collection of such additional information is warranted.

The measurement of treatment adherence likely involves the combination of measuring both levels of ASMs and seizure frequency. The goal of pharmacotherapy is to achieve seizure freedom, ideally without adverse side effects as well as consequently reduce morbidity and mortality and improve the quality of life of the person with epilepsy [3,37]. Interestingly, we found a strong correlation between self-reported ASM use and seizure frequency, with those experiencing more frequent seizures more likely to report taking ASM. This gradient was not as pronounced when determining adherence by detecting ASMs in the blood. This suggests that either individuals who self-reported as adherent were actually not taking their ASM as prescribed (resulting in the sub-optimal levels of ASM measured in the blood) or there are individual pharmacokinetic issues resulting in sub-optimal ASM blood levels when the participant is taking their ASM as prescribed. Understanding the timing of drug administration and drug pharmacokinetics, as noted above, are important considerations for future work.

We utilized Andersen’s behavior model [17] to examine possible factors associated with adherence. Individual beliefs are likely to impact on health-seeking and drug adherence. In the case of children, who are often dependent on caregivers, it is the caregiver’s beliefs that impact of health-seeking behavior. In this study, caregivers responded on behalf of child participants. However, caregiver beliefs were not explicitly explored. Further qualitative work aimed at more fully understanding the impact of beliefs, and specifically caregiver beliefs, on health-seeking behavior is warranted and would likely add to our understanding of the impact of beliefs on health-seeking behavior in rural South Africa.

Finally, another limitation of the current study is that it only explores convulsive epilepsy, which generally is more evident than other epilepsies. It is possible that people with less conspicuous seizure types, such as absence seizures and focal seizures without convulsions, are not as readily identified and diagnosed in this context and the cascade of care patterns for these other types of epilepsy may differ substantially.

## 5 Conclusion

This study provides the first attempt at presenting a care cascade for epilepsy in a rural sub-Saharan African setting. We have found that the majority people with ACE in rural South Africa have been told of their condition and the majority on individuals report receiving some level of pharmacological treatment. Of concern, non-adherence amongst rural South Africans is high, particularly amongst children. The specificity of self-reported of ASM use is low; suggesting further education by health care providers is warranted when delivering epilepsy care. A number of factors of perceived need were found to be significantly associated with higher levels of self-reported ASM usage in adults and children suggesting that increased patient education focused on the importance of ASM use may reduce non-adherence in the rural South African context.

## Funding

RGW gratefully acknowledges the EU Marie-Curie International Research Staff Exchange Scheme (grant no. 295168) and the South African National Research Foundation (119234) that allowed him to undertake and complete this work. Charles Newton was funded by the Wellcome Trust, UK (083744). The Agincourt HDSS is funded by the Wellcome Trust, UK (grants 058893/Z/99/A; 069683/Z/02/Z; 085477/Z/08/Z), with important contributions from the 10.13039/100009467University of the Witwatersrand, the 10.13039/501100001322South African Medical Research Council, and National Institute on Aging (NIA) of the National Institutes of Health (NIH).

## Declaration of Competing Interest

None of the authors have any conflict of interest to disclose.
