# Supplementary material for: Epilepsy care cascade, treatment gap and its determinants in rural South Africa
Source: Seizure. 2020 Aug;80:175–80. doi: 10.1016/j.seizure.2020.06.013 (PMC7443697; doi:10.1016/j.seizure.2020.06.013)
Supplement: Supplementary file 1 [file mmc1.docx]

**Table S1** Univariate analysis of factors associated with self-reported ASM use in adults (those 18 years and older)

| **Variable of Interest** | **No self-reported treatment** | **Self-reported treatment** | **Odds Ratios (95%CI)** | **p-values** |
| --- | --- | --- | --- | --- |
| **Predisposing Factors** |  |  |  |  |
| **Sex** |  |  |  |  |
| Female | 28 (26) | 80 (74) | . | . |
| Male | 27 (28) | 70 (72) | 0.91 (0.49-1.68) | 0.758 |
| **Ethnicity** |  |  |  |  |
| Mozambican origin | 13 (23) | 43 (77) | . | . |
| South African origin | 42 (28) | 106 (72) | 0.76 (0.37-1.56) | 0.459 |
| **Number of months present during previous year** | | |  |  |
| 0-6 months | 5 (38) | 8 (61) | . | . |
| 7-12 months | 50 (26) | 142 (74) | 1.78 (0.55-5.68) | 0.334 |
| **Belonging to Recognized Religion** | |  |  |  |
| No | 12 (30) | 28 (70) | . | . |
| Yes | 42 (26) | 121 (74) | 1.23 (0.58-2.65) | 0.588 |
| **Socio-economic Status (2007)** | |  |  |  |
| 1st quintile | 8 (25) | 24 (75) | . | . |
| 2nd quintile | 16 (36) | 29 (64) | 0.60 (0.22-1.65) | 0.326 |
| 3rd quintile | 12 (27) | 32 (73) | 0.89 (0.31-2.51) | 0.824 |
| 4th quintile | 11 (31) | 25 (69) | 0.76 (0.26-2.21) | 0.611 |
| 5th quintile | 7 (19) | 29 (81) | 1.38 (0.43-4.36) | 0.582 |
| **Previous use of traditional medicine** | |  |  |  |
| No | 16 (26) | 46 (74) | . | . |
| Yes | 26 (23) | 85 (77) | 1.14 (0.55-2.33) | 0.726 |
| **Union Status** |  |  |  |  |
| Never Married | 25 (27) | 68 (73) | . | . |
| Married | 12 (22) | 43 (78) | 1.32 (0.60-2.89) | 0.492 |
| Separated, Divorced, Widowed | 17 (31) | 39 (69) | 0.82 (0.39-1.71) | 0.600 |
| **Currently employed?** |  |  |  |  |
| No | 44 (26) | 123 (74) | . | . |
| Yes | 8 (30) | 18 (69) | 0.80 (0.33-1.98) | 0.637 |
| **Education (in years)** |  |  |  |  |
| None (0 years) | 11 (19) | 46 (81) | . | . |
| Primary (1-8 years) | 18 (25) | 54 (75) | 0.72 (0.31-1.67) | 0.442 |
| Secondary & Tertiary | 26 (34) | 50 (66) | 0.45 (0.20-1.03) | **0.060** |
| **Enabling/Impeding Factors** |  |  |  |  |
| **Distance from Dwelling to nearest primary health facility** | | | |  |
| <5km | 42 (25) | 126 (75) | . | . |
| 5km of more | 13 (35) | 24 (65) | 0.62 (0.29-1.32) | **0.211** |
| **Distance from Dwelling to nearest hospital** | | |  |  |
| 0-15km | 23 (24) | 72 (76) | **.** | **.** |
| >15km | 32 (29) | 78 (71) | 0.78 (0.42-1.45) | 0.432 |
| **Co-resident Kin availability** |  |  |  |  |
| *Co-resident with spouse* |  |  |  |  |
| No | 45 (27) | 120 (73) | . | . |
| Yes | 10 (25) | 30 (75) | 1.12 (0.51-2.49) | 0.771 |
| *Co-resident with mother* |  |  |  |  |
| No | 38 (32) | 81 (68) | . | . |
| Yes | 16 (21) | 61 (79) | 1.79(0.91-3.50) | **0.090** |
| *Number of co-resident household members* | |  |  |  |
| 0-1 members | 16 (36) | 28 (64) | . | . |
| 2-5 members | 21 (23) | 72 (77) | 1.96 (0.90-4.29) | **0.092** |
| 6-10 members | 15 (30) | 35 (70) | 1.33 (5.63-3.16) | 0.513 |
| >10 members | 3 (17) | 15 (83) | 2.86 (0.72-11.40) | **0.137** |
| **Perceived Need** |  |  |  |  |
| **Number of years with epilepsy** | |  |  |  |
| <1 year | 10 (71) | 4 (29) | . | . |
| 1-9 years | 18 (33) | 37 (67) | 5.14 (1.42-18.65) | **0.013** |
| 10-20 years | 9 (20) | 36 (80) | 10.0 (2.54-39.37) | **0.001** |
| >20 years | 18 (20) | 73 (80) | 10.14 (2.85-36.1) | **<0.001** |
| **Seizure Frequency** |  |  |  |  |
| Daily, Weekly, Monthly | 24 (47) | 27 (53) | 3.87 (1.95-7.69) | **<0.001** |
| Yearly | 28 (19) | 122 (81) | . | . |
| **Number of types of seizures** | |  |  |  |
| 1 type | 50 (29) | 123 (71) | . | . |
| >1 type | 5 (16) | 27 (84) | 2.20 (0.80-6.02) | **0.127** |
| **Self-report type of AED treatment** | |  |  | |
| Monotherapy | 79 (100) | 0 | . | . |
| Polytherapy | 51 (100) | 0 | . | . |
| Unknown | 20 (100) | 0 | . | . |
| **Previous hospitalization** |  |  |  | |
| No | 40 (27) | 107 (73) | . | . |
| Yes | 14 (25) | 42 (75) | 1.12 (0.55-2.27) | 0.750 |
| **Presence of burns** |  |  |  | |
| No | 45 (30) | 105 (70) | . | . |
| Yes | 5 (12) | 38 (88) | 3.26 (1.20-8.82) | **0.020** |
| **Learning difficulties** |  |  |  | |
| No | 41 (30) | 98 (71) | . | . |
| Yes | 10 (17) | 49 (83) | 2.05 (0.95-4.43) | **0.068** |
| **Neurological deficits** |  |  |  | |
| No | 42 (27) | 112 (73) | . | . |
| Yes | 9 (21) | 34 (79) | 1.42 (0.63-3.20) | 0.403 |
