# Supplementary material for: Epilepsy care cascade, treatment gap and its determinants in rural South Africa
Source: Seizure. 2020 Aug;80:175–80. doi: 10.1016/j.seizure.2020.06.013 (PMC7443697; doi:10.1016/j.seizure.2020.06.013)
Supplement: Supplementary file 2 [file mmc2.docx]

**Table S2** Univariate analysis of factors associated with ASM adherence in adults (those 18 years and older)

| **Variable of Interest** | **Not adherent** | **Adherent** | **Odds Ratios (95%CI)** | **p-values** |
| --- | --- | --- | --- | --- |
| **Predisposing Factors** |  |  |  |  |
| **Sex** |  |  |  |  |
| Female | 35 (56) | 27 (44) | . | . |
| Male | 41 (59) | 29 (41) | 0.92 (0.45-1.83) | 0.806 |
| **Ethnicity** |  |  |  |  |
| Mozambican origin | 24 (65) | 13 (35) | . | . |
| South African origin | 52 (55) | 43 (45) | 1.53 (0.70-3.35) | 0.292 |
| **Number of months present during previous 12** | | |  |  |
| 0-6 months | 2 (50) | 2 (50) | . | . |
| 7-12 months | 74 (58) | 54 (42) | 0.72 (0.14-7.10) | 0.756 |
| **Belonging to Organized Religion** | |  |  |  |
| No | 14 (58) | 10 (42) | . | . |
| Yes | 62 (57) | 46 (43) | 1.04 (-.42-2.55) | 0.934 |
| **Socio-economic Status (2007)** |  |  |  |  |
| 1st quintile | 11 (73) | 4 (27) | . | . |
| 2nd quintile | 18 (58) | 13 (42) | 1.99 (0.52-7.64) | 0.319 |
| 3rd quintile | 13 (46) | 15 (54) | 3.17 (0.81-12.4) | **0.097** |
| 4th quintile | 12 (55) | 10 (45) | 2.29 (0.55-9.47) | 0.252 |
| 5th quintile | 17 (61) | 11 (39) | 1.78 (0.45-7.02) | 0.411 |
| **Previous use of traditional medicine** | |  |  |  |
| No | 20 (61) | 13 (39) | . | . |
| Yes | 45 (59) | 31 (41) | 1.06 (0.50-2.44) | 0.891 |
| **Union Status** |  |  |  |  |
| Never Married | 33 (58) | 24 (42) | . | . |
| Married | 23 (66) | 12 (34) | 0.72 (0.30-1.72) | 0.456 |
| Separated, Divorced, Widowed | 20 (50 | 20 (50) | 1.38 (0.61-3.10) | 0.443 |
| **Currently employed?** |  |  |  |  |
| No | 69 (58) | 50 (42) | . | . |
| Yes | 6 (67) | 3 (33) | 0.69 (0.16-2.89) | 0.612 |
| **Education (in years)** |  |  |  |  |
| None (0 years) | 25 (63) | 15 (38) | . | . |
| Primary (1-8 years) | 29 (54) | 25 (46) | 1.44 (0.62-3.31) | 0.395 |
| Secondary & Tertiary | 22 (58) | 16 (42) | 1.21 (0.48-3.00) | 0.678 |
| **Enabling/Impeding Factors** |  |  |  |  |
| **Distance from Dwelling to nearest primary health facility** | | | |  |
| <5km | 62 (58) | 45 (42) | . | . |
| 5km of more | 14 (56) | 11 (44) | 1.08 (0.45-2.60) | 0.859 |
| **Distance from Dwelling to nearest hospital** | | |  |  |
| 0-15km | 32 (62) | 20 (38) | **.** | **.** |
| >15km | 44 (55) | 36 (45) | 1.31 (0.64-2.67) | 0.458 |
| **Coresident Kin availability** |  |  |  |  |
| *Coresident with spouse* |  |  |  |  |
| No | 61 (55) | 49 (44) | . | . |
| Yes | 15 (68) | 7 (41) | 0.58 (0.22-1.54) | 0.274 |
| *Coresident with mother* |  |  |  |  |
| No | 50 (63) | 29 (37) | . | . |
| Yes | 25 (51) | 24 (49) | 1.66 (0.80-3.41) | **0.172** |
| *Number of co-resident household members* | |  |  |  |
| 0-1 members | 11 (46) | 13 (54) | . | . |
| 2-5 members | 38 (62) | 23 (38) | 0.51 (0.20-1.33) | **0.170** |
| 6-10 members | 19 (54) | 16 (46) | 0.71 (0.25-2.02) | 0.520 |
| >10 members | 8 (67) | 4 (33) | 0.42 (0.10-1.79) | **0.243** |
| **Perceived Need** |  |  |  |  |
| **Number of years with epilepsy** | |  |  |  |
| <1 year | 7 (78) | 2 (22) | . | . |
| 1-9 years | 28 (70) | 12 (30) | 1.5 (0.27-8.30) | 0.620 |
| 10-20 years | 10 (40) | 15 (60) | 5.25 (0.90-30.62) | **0.065** |
| >20 years | 31 (53) | 27 (47) | 3.05 (0.58-15.94) | 0.187 |
| **Seizure Frequency** |  |  |  |  |
| Daily, Weekly, Monthly | 55 (60) | 37 (40) | 0.75 (0.35-1.60) | 0.454 |
| Yearly | 20 (53) | 18 (47) | . | . |
| **Number of types of seizures** |  |  |  |  |
| 1 type | 65 (60) | 44 (40) | . | . |
| >1 type | 11 (48) | 12 (52) | 1.61 (0.65-3.98) | 0.300 |
| **Self-report type of AED treatment** | |  |  | |
| Monotherapy | 23 (42) | 31 (57) | . | . |
| Polytherapy | 20 (57) | 15 (43) | 0.55 (0.24-1.31) | **0.181** |
| Unknown | 2 (33) | 4 (66) | 1.48 (0.25-8.80) | 0.664 |
| **Previous hospitalization** |  |  |  | |
| No | 60 (59) | 42 (41) | . | . |
| Yes | 15 (52) | 14 (48) | 1.33 (0.58-3.05) | 0.496 |
| **Presence of burns** |  |  |  | |
| No | 60 (59) | 42 (41) | . | . |
| Yes | 15 (54) | 13 (46) | 1.24 (0.53-2.87) | 0.619 |
| **Learning difficulties** |  |  |  | |
| No | 54 (61) | 34 (39) | . | . |
| Yes | 21 (49) | 22 (51) | 1.66 (0.80-3.47) | **0.175** |
| **Neurological deficits** |  |  |  | |
| No | 60 (60) | 40 (40) | . | . |
| Yes | 15 (50) | 15 (50) | 1.50 (0.66-3.41) | 0.332 |
