# Supplementary material for: Epilepsy care cascade, treatment gap and its determinants in rural South Africa
Source: Seizure. 2020 Aug;80:175–80. doi: 10.1016/j.seizure.2020.06.013 (PMC7443697; doi:10.1016/j.seizure.2020.06.013)
Supplement: Supplementary file 3 [file mmc3.docx]

**Table S3** Univariate analysis of factors associated with self-reported ASM use in children (younger than 18 years)

| **Variable of Interest** | **No self-reported treatment** | **Self-reported treatment** | **Odds Ratios (95%CI)** | **p-values** |
| --- | --- | --- | --- | --- |
| **Predisposing Factors** |  |  |  |  |
| **Sex** |  |  |  |  |
| Female | 20 (56) | 16 (44) | . | . |
| Male | 31 (61) | 20 (39) | 0.81 (0.34-1.92) | 0.626 |
| **Ethnicity** |  |  |  |  |
| Mozambican origin | 19 (66) | 10 (34) | . | . |
| South African origin | 32 (55) | 26 (45) | 1.54 (0.61-3.89) | 0.357 |
| **Number of months present during previous 12** | | |  |  |
| 0-6 months | 1 (33) | 2 (67) | . | . |
| 7-12 months | 50 (60) | 34 (40) | 0.34 (0.03-3.90) | 0.386 |
| **Belonging to Organized Religion** | |  |  |  |
| No | 1 (25) | 3 (75) | . | . |
| Yes | 46 (67) | 23 (33) | 0.17 (0.02-1.69) | **0.130** |
| **Socio-economic Status (2007)** | |  |  |  |
| 1st quintile | 6 (67) | 3 (33) | . | . |
| 2nd quintile | 16 (73) | 6 (27) | 0.75 (0.14-4.00) | 0.736 |
| 3rd quintile | 12 (55) | 10 (45) | 1.67 (0.33-8.42) | 0.527 |
| 4th quintile | 10 (67) | 5 (33) | 1.00 (0.17-5.77) | 1 |
| 5th quintile | 7 (50) | 7 (50) | 2.00 (0.35-11.36) | 0.434 |
| **Previous use of traditional medicine** | |  |  |  |
| No | 10 (53) | 9 (47) | . | . |
| Yes | 22 (63) | 13 (37) | 0.66 (0.21-2.04) | 0.466 |
| **Mother's Union Status** |  |  |  |  |
| Never Married | 17 (55) | 14 (45) | . | . |
| Married | 22 (69) | 10 (31) | 0.55 (0.20-1.54) | 0.258 |
| Separated, Divorced, Widowed | 7 (70) | 3 (30) | 0.52 (0.11-2.39) | 0.402 |
| **Mother currently employed?** | |  |  |  |
| No | 34 (63) | 20 (37) | . | . |
| Yes | 13 (68) | 6 (32) | 0.78 (0.26-2.39) | 0.67 |
| **Mother's Education (in years)** | |  |  |  |
| None (0 years) | 0 | 0 | . | . |
| Primary (1-8 years) | 43 (64) | 24 (35) | . | . |
| Secondary & Tertiary | 0 | 0 | . | . |
| **Enabling/Impeding Factors** |  |  |  |  |
| **Distance from Dwelling to nearest primary health facility** | | | |  |
| <5km | 41 (55) | 33 (45) | . | . |
| 5km of more | 10 (77) | 3 (23) | 0.37 (0.09-1.47) | **0.158** |
| **Distance from Dwelling to nearest hospital** | | |  |  |
| 0-15km | 17 (46) | 20 (54) | **.** | **.** |
| >15km | 34 (68) | 16 (32) | 0.4 (0.17-0.96) | **0.041** |
| **Coresident Kin availability** |  |  |  |  |
| *Coresident with mother* |  |  |  |  |
| No | 9 (56) | 7 (44) | . | . |
| Yes | 40 (58) | 29 (42) | 0.93 (0.31-2.79) | 0.900 |
| *Number of co-resident household members* | |  |  |  |
| 0-1 members | 18 (58) | 13 (42) | . | . |
| 2-5 members | 26 (59) | 18 (41) | 0.96 (0.38-2.44) | 0.929 |
| 6-10 members | 6 (55) | 5 (45) | 1.15 (0.29-4.61) | 0.839 |
| >10 members | 1 (100) | 0 | . | . |
| **Perceived Need** |  |  |  |  |
| **Number of years with epilepsy** | |  |  |  |
| <1 year | 11 (85) | 2 (15) | . | . |
| 1-9 years | 27 (64) | 15 (36) | 3.05 (0.60-15.65) | **0.18** |
| 10-20 years | 5 (29) | 12 (71) | 13.2 (2.11-82.50) | **0.006** |
| >20 years | 8 (53) | 7 (47) | 4.81 (0.78-29.59) | **0.090** |
| **Seizure Frequency** |  |  |  |  |
| Daily, Weekly, Monthly | 16 (38) | 26 (62) | 6.32 (2.42-16.5) | **<0.001** |
| Yearly | 35 (80) | 9 (20) | . | . |
| **Number of types of seizures** | |  |  |  |
| 1 type | 41 (62) | 25 (38) | . | . |
| >1 type | 10 (50) | 10 (50) | 1.64 (0.60-4.49) | 0.336 |
| **Self-report type of AED treatment** | |  |  | |
| Monotherapy |  |  | . |  |
| Polytherapy |  |  | . |  |
| Unknown |  |  | . |  |
| **Previous hospitalization** |  |  |  | |
| No | 43 (59) | 30 (41) | . |  |
| Yes | 7 (54) | 6 (46) | 1.23 (0.38-4.02) | 0.340 |
| **Presence of burns** |  |  |  | |
| No | 48 (60) | 32 (40) | . | . |
| Yes | 0 | 4 (100) | . | . |
| **Learning difficulties** |  |  |  | |
| No | 45 (67) | 22 (33) | . | . |
| Yes | 5 (26) | 14 (74) | 5.73 (1.83-17.93) | **0.003** |
| **Neurological deficits** |  |  |  | |
| No | 47 (64) | 27 (36) | . | . |
| Yes | 3 (27) | 8 (73) | 4.64 (1.13-19.00) | **0.033** |
