# Supplementary material for: Epilepsy care cascade, treatment gap and its determinants in rural South Africa
Source: Seizure. 2020 Aug;80:175–80. doi: 10.1016/j.seizure.2020.06.013 (PMC7443697; doi:10.1016/j.seizure.2020.06.013)
Supplement: Supplementary file 4 [file mmc4.docx]

**Table S4** Univariate analysis of factors associated with ASM adherence in children (younger than 18 years)

| **Variable of Interest** | **Not adherent** | **Adherent** | **Odds Ratios (95%CI)** | **p-values** |
| --- | --- | --- | --- | --- |
| **Predisposing Factors** |  |  |  |  |
| **Sex** |  |  |  |  |
| Female | 17 (81) | 4 (19) | . | . |
| Male | 22 (76) | 7 (24) | 1.35 (0.34-5.39) | 0.669 |
| **Ethnicity** |  |  |  |  |
| Mozambican origin | 13 (81) | 3 (19) | . | . |
| South African origin | 26 (76) | 8 (24) | 1.33 (0.30-5.88) | 0.704 |
| **Number of months present during previous 12** | | | |  |
| 0-6 months | 2 (100) | 0 | . | . |
| 7-12 months | 77 (37) | 11 (23) | . | . |
| **Belonging to Organized Religion** | |  |  |  |
| No | 0 | 1 (100) | . | . |
| Yes | 33 (83) | 7 (18) | . | . |
| **Socio-economic Status (2007)** | |  |  |  |
| 1st quintile | 5 (83) | 1 (17) | . | . |
| 2nd quintile | 8 (73) | 3 (27) | 1.88 (0.15-23.40) | 0.625 |
| 3rd quintile | 12 (71) | 5 (29) | 2.08 (0.19-22.67) | 0.547 |
| 4th quintile | 8 (100) | 0 | . | . |
| 5th quintile | 4 (67) | 2 (33) | 2.50 (0.16-38.60) | 0.512 |
| **Previous use of traditional medicine** | | |  |  |
| No | 6 (67) | 3 (33) | . | . |
| Yes | 14 (74) | 5 (26) | 0.71 (0.13-399) | 0.702 |
| **Mother's union Status** | |  |  |  |
| Never Married | 14 (74) | 5 (26) | . | . |
| Married | 14 (82) | 3 (18) | 0.60 (0.12-3.01) | 0.534 |
| Separated, Divorced, Widowed | 5 (83) | 1 (17) | 0.56 (0.05-6.03) | 0.633 |
| **Mother currently employed?** | |  |  |  |
| No | 25 (78) | 7 (22) | . | . |
| Yes | 8 (80) | 2 (20) | 0.89 (0.15-5.20) | 0.900 |
| **Mother's education (in years)** | |  |  |  |
| None (0 years) | 0 | 0 | . | . |
| Primary (1-8 years) | 28 (80) | 7 (20) | . | . |
| Secondary & Tertiary | 0 | 0 | . | . |
| **Enabling/Impeding Factors** | |  |  |  |
| **Distance from Dwelling to nearest primary health facility** | | | |  |
| <5km | 31 (76) | 10 (24) | . | . |
| 5km of more | 8 (89) | 1 (11) | 0.39 (0.04-3.49) | 0.398 |
| **Distance from Dwelling to nearest hospital** | | |  |  |
| 0-15km | 15 (68) | 7 (32) | **.** | **.** |
| >15km | 24 (86) | 4 (14) | 0.36 (0.09-1.43) | **0.146** |
| **Coresident Kin availability** | |  |  |  |
| *Coresident with mother* |  |  |  |  |
| No | 6 (67) | 3 (33) | . | . |
| Yes | 32 (80) | 8 (20) | 0.50 (0.10-2.45) | 0.392 |
| *Number of co-resident household members* | | |  |  |
| 0-1 members | 15 (75) | 5 (25) | . | . |
| 2-5 members | 18 (78) | 5 (22) | 0.83 (0.20-3.44) |  |
| 6-10 members | 6 (86) | 1 (14) | 0.50 (0.05-5.22) |  |
| >10 members | 0 | 0 | . | . |
| **Perceived Need** |  |  |  |  |
| **Number of years with epilepsy** | |  |  |  |
| <1 year | 5 (83) | 1 (17) | . | . |
| 1-9 years | 21 (75) | 7 (25) | 1.67 (0.17-16.81) | 0.665 |
| 10-20 years | 8 (73) | 3 (27) | 1.88 (0.15-23.40) | 0.625 |
| >20 years | 5 (100) | 0 | . | . |
| **Seizure Frequency** |  |  |  |  |
| Daily, Weekly, Monthly | 22 (88) | 3 (12) | 3.67 (0.84-16.03) | **0.084** |
| Yearly | 16 (67) | 8 (33) | . | . |
| **Number of types of seizures** | |  |  |  |
| 1 type | 27 (77) | 8 (23) | . | . |
| >1 type | 12 (80) | 3 (20) | 0.84 (0.19-3.75) | 0.823 |
| **Self-report type of AED treatment** | |  |  | |
| Monotherapy |  |  | . |  |
| Polytherapy |  |  |  |  |
| Unknown |  |  |  |  |
| **Previous hospitalization** | |  |  | |
| No | 36 (82) | 8 (18) | . | . |
| Yes | 2 (40) | 3 (60) | 6.75 (0.96-47.27) | **0.054** |
| **Presence of burns** |  |  |  | |
| No | 38 (79) | 10 (21) | . | . |
| Yes | 0 | 1 (100) | . | . |
| **Learning difficulties** |  |  |  | |
| No | 34 (81) | 8 (19) | . | . |
| Yes | 5 (63) | 3 (38) | 2.55 (0.50-12.96) | 0.259 |
| **Neurological deficits** |  |  |  | |
| No | 36 (80) | 9 (20) | . | . |
| Yes | 3 (60) | 2 (40) | 2.67 (0.39-18.4) | 0.320 |
